# An Animation Model Generation Method Based on Gaussian Mutation Genetic Algorithm to Optimize Neural Network

**DOI:** 10.1155/2022/5106942

**Published:** 2022-06-03

**Authors:** Jing Liu, Qixing Chen, Yihua Zhang, Xiaoying Tian

**Affiliations:** ^1^College of Culture and Art, Chengdu University of Information Technology, Chengdu 610225, China; ^2^College of Communication Engineering, Chengdu University of Information Technology, Chengdu 610225, China; ^3^Library, Chengdu University of Information Technology, Chengdu 610225, China

## Abstract

With the rapid development of computer graphics, 3D animation has been applied to all fields of people's lives, especially in the industries of film and television works, games, and entertainment. The wide application of animation technology makes it difficult for general 3D animation effects to impress increasingly discerning audiences. Group animation, as a new focus, has received more and more attention and has become a hot issue in computer graphics. Traditional animation production mainly relies on manual drawing and key frame technologies. The limitations of these technologies make the production of group animation consume a lot of manpower, financial resources, and time, and cannot guarantee the intelligence of characters and the authenticity of group behavior. Therefore, in order to end the above issues, this paper proposes an animation model generation method based on Gaussian mutation genetic algorithm to optimize neural network, including obtaining animation scene data, according to the animation scene data, and extracting animation model elements. The elements are input into the model network, the target animation model is generated, and the target animation model is displayed. The method proposed in this paper improves the animation model generation method in the prior art to a certain extent. The proposed animation model is constructed only through fixed rules, and the composition rules of the model cannot be changed according to the historical data of the animation model construction and other factors. Technical issues that reduce the flexibility and accuracy of the animation model generation.

## 1. Introduction 

With the continuous deepening of related researches on computer graphics and 3D animation technology, the use of 3D special effects in the fields of film and television works and game entertainment is not uncommon. The widespread application of animation technology makes it difficult for ordinary three-dimensional animation effects to impress an increasingly discerning audience. Animators began to work on finding more novel special effects production methods. Crowd animation production methods have been continuously developed and improved, and the demand for special effects in film and television is one of the important reasons for its development. At present, the mass animation production method has been widely used in many commercial movies, and commercial software specially used for mass animation production has been produced, such as the famous large-scale mass animation software, which created amazing animations in the movie “The Lord of the Rings,” and visual effects and won the 2003 Academy Award for Science and Engineering. In addition to film and television effects, crowd animation is widely used in the field of game entertainment and simulation research, so crowd animation has profound research significance [1–8].

**Figure 1:**
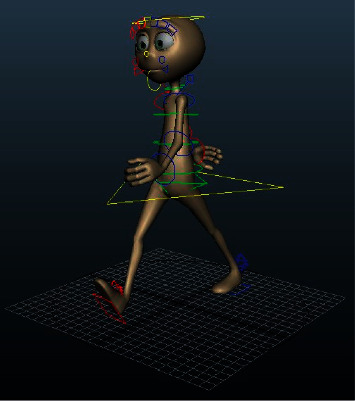
3D animation production.

Traditional 3D animation production methods (as shown in Figure 1) are generally divided into two types: one is to use a camera to shoot, and the other is to use computer-related technologies [9]. Due to the limitations of hardware, the traditional shooting method has become smaller and smaller. The traditional group animation production method has produced many excellent animations, but it has some unavoidable limitations, such as (1) It is difficult for individual modeling to achieve diversity. The modeling of animated characters is designed by the animator's step by step from the sketch. The construction of the model requires the imagination and creativity of the animator and the design time of a single modeling is long and inefficient. (2) The tedious action details are depicted. Every action of an animated character needs to be planned one by one by an animator, an animator needs to control every detail of an animated character, and a lot of energy of an animator is spent on this low-level control. (3) Animated characters lack autonomy. The behavior of an animated character is affected by the environment in which it is located. A slight modification of the animation script requires the entire animation to be remade and the character cannot automatically coordinate with its surrounding environment. Characters driven by keyframe or motion data methods lack autonomy, reducing animation modifiability and interactivity.

In order to make the group behavior meet the authenticity and regularity of the overall movement, and make the individual ensure its own independence and sociality. Related researches have been focused on two aspects: one is the reality of behavior, and the other is the realistic embodiment of groups in virtual environments. For this reason, research on group behavior animation based on intelligent optimization algorithm has been produced one after another. For example, genetic algorithm is used in 3D character modeling, and the improved particle swarm algorithm is used to plan the path of the group, which improved the production efficiency of group animation. The worker bee colony algorithm is used to simulate the group diffusion behavior and the psychological factors when the group chooses the destination is added to the fitness function, which truly reflects the natural law of group movement and has a good application prospect.

With the great improvement of computing speed and the popularization of parallel computing, the computational speed bottleneck of genetic algorithm has disappeared [10–16]. People gradually pay more attention to some basic problems of genetic algorithm, and many researchers have proposed improved genetic algorithms, such as coevolutionary algorithm, decentralized parallel genetic algorithm, adaptive genetic algorithm, and so on. Genetic algorithms have achieved success in machine learning, process control, economic forecasting, engineering optimization, etc., and have attracted extreme attention from experts in economics, and engineering applications—great interest.

Research on genetic algorithms and evolutionary computing has been on the rise, and many monographs have been published one after another. Especially in recent years, related applications have achieved remarkable results in many fields, and related monographs and discussion websites are also increasing [17–20]. The local search ability of Gaussian mutation is better and thus it can be used to optimize the complicated problem.

Although the genetic algorithm has many advantages, it also has many problems. For example, there are many ways to calibrate the fitness value, which is prone to the phenomenon of “prematurity.” The closer it is to the optimal solution, the easier it is to swing around the optimal solution, and the convergence speed is also slower. Therefore, this paper proposes a hybrid genetic algorithm with neural networks based on Gaussian mutation for these problems. That is, on the basis of the Gaussian mutation genetic algorithm, a hill-climbing operator is added to strengthen the local search ability, so that the algorithm can find the Pareto optimal set more quickly and accurately.

## 2. Gaussian Mutation Genetic Algorithm to Optimize Neural Networks

Therefore, before using the genetic algorithm, the mapping from phenotype to genotype, that is, coding, needs to be realized. The encoding methods mainly include binary encoding, real number encoding, and so on.

**Figure 2:**
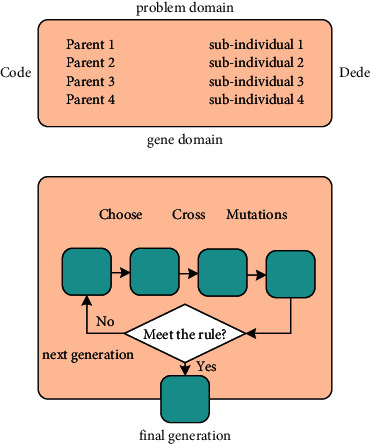
The general process of a basic genetic algorithm.

Figure 2 shows the general process of a basic genetic algorithm. This paper takes an example in the animation as the research object and optimizes the animation path generation method based on the Gaussian mutation genetic algorithm to optimize neural networks. Neural network has been widely used to a lot of fields, and too many different neural networks have been proposed and achieved good results [21–32].

First, set the number of iterative evolutionary populations. In the simulation experiment based on PYOPENGL, the number of each generation of evolution is set to 400. After the parameter adjustment of the algorithm in the later stage, the termination is set. The number of iterations is 300 generations, that is, it automatically finds the way to start timing, and can travel 170 steps.

Then use python to design and program this algorithm and use python to establish two categories of Lines and Rectangles. The starting point is a certain point coordinate set in the two-dimensional plane coordinate system established in OpenGL, and the end point judgment function is set at the end point of the algorithm. In this track, a line segment is displayed. Once it touches the line segment, that is, it is deemed that the end point can be reached.

The connection of any neuron in the BP neural network is used in this paper. The schematic diagram of input and output *m* is the number of inputs corresponding to the nodes. The calculation principle is that the weight *w* matrix:

(1) $$\begin{array}{c} \left(w_{1},w_{2},\ldots ,w_{m}\right). \end{array}$$

**Figure 3:**
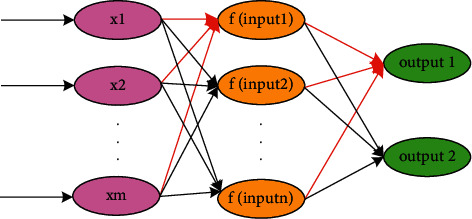
BP feedforward neural network.

The threshold *b* matrix is matrix multiplied with the input matrix, and the obtained matrix is summed, which is the equation (2):

(2) $$\begin{array}{c} \left(b_{1},b_{2},\ldots ,b_{m}\right),\;\text{input}=\sum_{i=1}^{m}\left(w_{i}x_{i}+b_{i}\right), \end{array}$$

where *x*_*i*_ refers to the input parameters of several neuron nodes in the input layer, that is, the input parameters corresponding to sensor *i*. As shown in Figure 3, the result of the matrix summation is passed to the activation function Sigmoid function, as shown in equation (3):

(3) $$\begin{array}{c} f=\frac{1}{1+\;\exp^{-\text{input}}}. \end{array}$$

Using a relatively simple Sigmoid function (Sigmoid function) as the activation function of each layer is beneficial to the convergence of the learning algorithm. The calculation method of a single neuron is extended to the topology of the entire neural network, as shown in Figure 3.

In Figure 3, the calculation from the input nodes *x*_1_, *x*_2_,…, *x*_*m*_ to the first node of the hidden layer is the calculation process of equations (2) and (3) and *f*(input 1) is the output of the hidden layer node. Assuming that the number of hidden layer nodes is *n* and the number of output layers is 1, the calculation process of equations (2), (3) will be performed *n∗*1 times, where the output calculation equation is (5). It should be noted that the symbol *∗* in this paper means the convolution rather than multiplication. Arbitrary output calculation is similar to (5), only the connection weights and thresholds are different. Each connection in the graph has its own connection weight and threshold. Considering that the threshold can be shared, for a third-layer classical neural network, the weight matrix dimension of the connection weight between the input layer and the hidden layer is *n∗m*, and the connection weight between the hidden layer and the output layer is *n∗m*. The dimension of the weight matrix is *n∗*1, and the dimensions of the corresponding threshold matrix are *n∗*1,1*∗*1, respectively.

(4) $$\begin{array}{c} \text{output}1=\frac{1}{1+\;\exp^{f\left(\text{input}1\right)+f\left(\text{input}2\right)+\cdots +f\left(\text{input}n\right)}}. \end{array}$$

The third step is to set the topology of the neural network. In the design of the automatic pathfinding algorithm, due to the excessive optimization parameters of the car, in order to avoid the complex structure of the neural network in the later stage and the cumbersome processing of information, due to the needs of research, the structure of the neural network. Due to the needs of research, it is tentatively designated as a classic three-layer feedforward neural network, input layer, hidden layer, and output layer. In the three-layer neural network, the number of neurons in the hidden layer is set to 15. Due to the input of seven parameters (the distance of the car sensor in five directions, and the current driving speed and current driving angle of the car), the input number *m* is 7. The output layer has two neuron nodes, that is, the two output parameters control the speed and driving angle of the car, respectively, according to their outputs. Then the number of weights *w*1 (the connection weight between the input layer and the hidden layer) is 15∗7 = 105. The number of threshold *b*1 is 15; the number of weights *w*2 (the connection weight between the hidden layer and the output layer) is 2∗15 = 30, the number of thresholds is 2, so the number of parameters to be optimized based on the Gaussian mutation genetic algorithm is 105 + 15 + 30 + 2 = 152, which belongs to the category of high-dimensional search and optimization problems, and has certain complexity.

In the fourth step, the continuous optimization process of the weights and thresholds of the neural network is cumbersome, and the coding requirement is to reflect the features of the solution as much as possible. Because the input variable parameters of the input layer used in conventional coding are all binary, similar to the coding form of 000111, considering the large number of car iteration populations in the algorithm design process, the length of chromosome coding is long, and finally needs to be decoded into real numbers again, so that the weights. The changes of the value and threshold cannot achieve random and continuous effects, which will eventually affect the speed of unsupervised learning of the car and the iterative evolution performance of the algorithm. Therefore, the programming of the neural network part of the algorithm is all encoded with real numbers. A total of 13 real numbers. The first bit represents the positive and negative bits. The real numbers are combined into a string array in the *w*1*biw*2*b* arrangement of the neural network for encoding and stored in the algorithm in a certain number index order. Initially determine the neural network matrix represented by the automatic pathfinding car population. The algorithm design requires that the neural output is to use the sigmoid function to calculate and output a 0–1 random real number, so as to control the bottom speed and driving angle of the car. The two output bottom-level decisions of the output layer are made according to the following equations (5) and (6). There is still a problem here. The output value of the neural network with the sigmoid function as the activation function is a number between 0 and 1, which cannot directly drive the movement of the car. Output 2, output 1 control the acceleration and deceleration of the car, output 2 controls the car to turn left and right, the specific provisions are the following:

(5) $$\begin{array}{c} \text{output}1\left\{\begin{array}{c} <0.3\text{ slow down}\\ <0.7\text{ speed up}\\ <1\text{ constant speed,} \end{array}\right. \end{array}$$

(6) $$\begin{array}{c} \text{output}2\left\{\begin{array}{c} <0.3\text{ turn left}\\ <0.7\text{ turn right}\\ <1\text{ go straight.} \end{array}\right. \end{array}$$

The fifth step is to set the fitness function. Here, set the time from the start of the car to the car to hit the wall as the survival time of the car t/0.01, that is, to evaluate the pros and cons of the evolution of the car. In this algorithm design, set the longer the survival time of the car, the higher the score of the car, the greater the driving distance of the car, and the easier it is to reach the end of the specified track.

The sixth step is to carry out the iterative loop process. It is mainly the iterative evolution process of the Gaussian mutation of the 400 cars of the genetic algorithm, and it is also the process of random automatic pathfinding of the cars in the track designed in the first step.

Gaussian mutation belongs to one of the mutation strategies of genetic algorithm. The normal distribution is a very important probability distribution in the fields of mathematical physics and engineering. The local search ability of Gaussian mutation is good, which is not conducive to global convergence.

(7) $$\begin{array}{c} f\left(x\right)=\frac{1}{\sigma \sqrt{2\pi }}e^{{\left(x-\mu \right)}^{2}/2\sigma^{2}}. \end{array}$$

Equation (7) is the probability density function of the Gaussian distribution. Standard normal distribution only needs to make *μ* = 0, *σ* = 1. The image of Gaussian distribution is a schematic diagram of randomly generated random numbers conforming to the standard Gaussian distribution. These random numbers perturb the four populations with hierarchical weights and thresholds in different proportions. On the basis of the existing genetic variable matrix, a matrix composed of random numbers conforming to the Gaussian distribution and having the same dimension as the genetic variable is added, so as to realize the local search near the original solution space. RANDN is a set of random number matrices conforming to Gaussian distribution:

(8) $$\begin{array}{c} x^{t+1}=x^{t}+\text{rand}n∼\left(\mu ,\sigma^{2}\right). \end{array}$$

In this paper, the arithmetic crossover operator is used, which generates new individuals by linear combination between two bodies. Assuming that the arithmetic crossover operation is performed on individuals, it is mainly divided into two steps: one is to determine the coefficients of the linear combination of individuals; the other is to generate new individuals according to equations (10)–(11). The new individuals generated after the crossover operation are the following:

(9) $$\begin{array}{c} X_{A}^{t+1}=\omega X_{B}^{t}+\left(1-\omega \right)X_{A}^{t}, \end{array}$$

(10) $$\begin{array}{c} X_{B}^{t+1}=\omega X_{A}^{t}+\left(1-\omega \right)X_{B}^{t}, \end{array}$$

where *ω* is a parameter and belongs to [0, 1].

Gaussian mutation consists of creating a descendant from a Gaussian distribution to each element of an individual vector adding a random value as follows:

(11) $$\begin{array}{c} y_{i}^{\left(t+1\right)}=x_{i}^{\left(t\right)}+kN\left(0,1\right). \end{array}$$

In the equation, *y*_*i*_ and *x*_*i*_ are the *i*-th gene of the new generation and parental individuals, respectively, *k* is the value set by the user, and *N* (0, 1) is a standard normal distribution.

## 3. Animation Model Generation Method Based on Gaussian Mutation Genetic Algorithm to Optimize Neural Network

**Figure 4:**
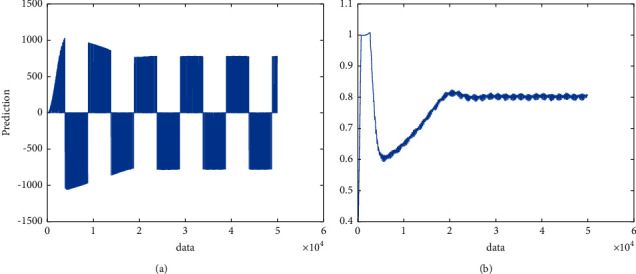
Predicted value comparison: (a) predicted value. (b) Variation.

Group movement is a very interesting phenomenon in nature. The large flocks of migratory birds flying south in the sky, the running of antelopes on land, and the movement of fish in the ocean are all very spectacular, and the momentum often makes us amazed. In the process of group movement, the behavior of each individual is independent, but at the same time, the movement of the whole group is a whole. When encountering danger, the individuals in the group fled in all directions. On the surface, it looks very chaotic, but there are also rules to follow—the original group is divided into several small groups and flees in different directions. The movement of individuals in a group seems to be random, but there is a certain movement law. On the one hand, individuals maintain a certain distance between them, and on the other hand, they also have roughly the same direction of movement. The entire group movement is based on each individual movement, and the individual adjusts his movement direction and various states through the perception of the environment and companions. The predicted value based on animation model generation method is shown in Figure 4.

In animation model generation method, it is often necessary to create large-scale group movements. The method of adding motion paths for each individual in the group individually can be tolerated by animators when the number of groups is small, but it is not feasible for thousands of individuals. Studying the law of group motion automatically generates a path with a certain degree of intelligence. After binding the model and the path, the group animation that moves according to the path can be generated. What the animator needs to control is only the macro properties of the group, such as controlling the overall movement direction of the group, adding random changes to each bound individual movement can create a group animation that is close to the desired effect, and the animator's workload is greatly reduced.

**Figure 5:**
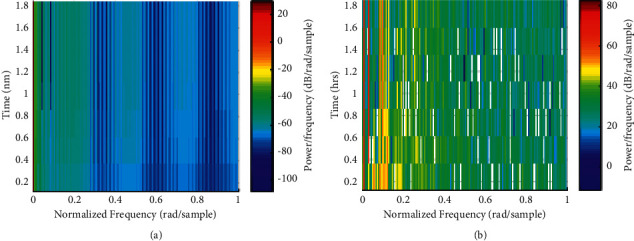
Normalized frequency comparison with or without optimization. (a) Condition 1. (b) Condition 2.

Path planning is to find an optimal (or suboptimal) motion path from a given starting point to an end point according to certain criteria (such as the shortest total length of the walking route, the least energy consumption, etc.) in an environment with obstacles. Go around all obstacles safely and without collision. Path planning mainly solves three problems: (1) The path can reach the target point from the initial point. (2) The obstacle can be circumvented by a certain algorithm path.; (3) The motion trajectory is optimized as much as possible. Path planning methods based on animation model generation method are mainly divided into two categories: one is the path planning method based on the environment model, and the other is the path planning method based on the behavior. The normalized frequency is shown in Figure 5.

The design standard of global path planning is to maximize the planning effect. There are many mature methods in this field, including the visual graph method, the tangent graph method, the Voronoi graph method, the topology method, and the grid method. The first four methods use a connection graph to represent the workspace, which is an idea based on graph theory. The grid method uses grids to describe the working environment, and then performs obstacle avoidance planning to obtain a collision-free path.

**Figure 6:**
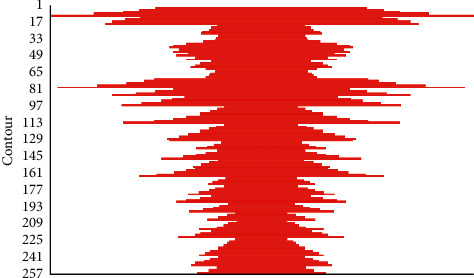
Contour of the results.

The main purpose of local path planning is to improve the ability to avoid obstacles, and the path optimization effect is the second. The first four methods are relatively widely used, while the latter ones have only become more popular in recent years.  Figure 6 shows the contour.

The behavior-based method decomposes the navigation problem into many relatively independent behavioral units. The behaviors adopted by each behavioral unit are different, and these units work in coordination with each other to complete the navigation task.

**Figure 7:**
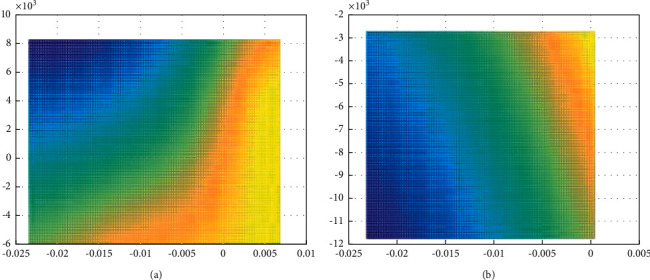
Evaluated results comparison with/without optimization: (a) optimized case. (b) Without optimization.

Animation model generation methods can be roughly divided into three types: reflective behavior, reactive behavior, and deliberative behavior. Reflexive behavior is a momentary irritable instinctive response that can respond quickly to unexpected situations. The method based on reactive behavior is to directly read sensor data to plan the next action, which can respond stably and timely to unpredictable obstacles and environmental changes, and is suitable for path planning of mobile robots in complex environments. Deliberate behavior uses the known global environment model to provide the optimal action sequence for the intelligent system to reach a specific goal, which is suitable for planning in complex static environments, but because deliberation planning requires a certain amount of time to execute, it cannot be used in the environment. Predicted changes respond more slowly.  Figure 7 shows the evaluated results.

The 3D environment modeling refers to processing the description of the motion environment from the original form into a suitable computer model through a series of methods, so as to realize the abstract representation of the environment. In path planning research, the abstract representation of the environment plays a very important role in the algorithm design. Environmental modeling includes two aspects: basic information of the environment and description of obstacles.

The grid method was proposed by Howden to use grids to represent maps when doing path planning. The specific method is to take the environmental area on a two-dimensional plane, the maximum length in the horizontal direction is *L*, the maximum width in the vertical direction is *W*, and the length and width of the grid are *b*, then the number of grids in each row is: *N*_*x*_ = *L*/*b*, the number of grids per column is *N*_*y*_ = *W*/*b*.

**Figure 8:**
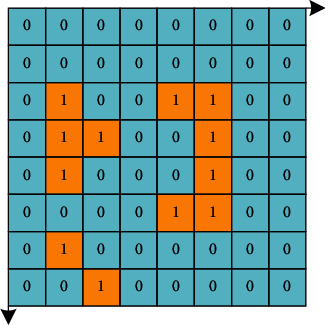
Grid method used in this paper.

Grids divided in two-dimensional space are usually identified by a rectangular coordinate system, as shown in Figure 8. For the three-dimensional environment, the grid is marked according to the three coordinate axes of *XYZ* and represented by several solid squares whose length, width and height are all 1.

**Figure 9:**
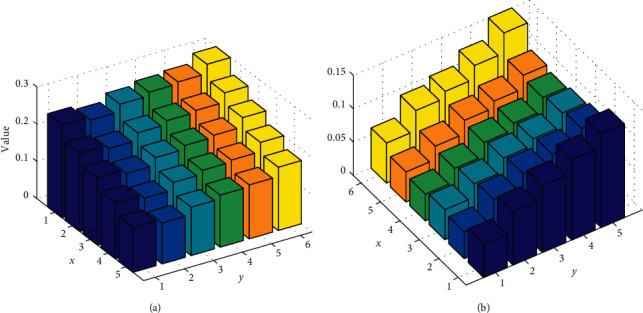
Result comparison before and after optimization: (a) after optimization. (b) Before optimization.

However, when modeling with the grid method and animation model generation method, there are usually more or less irregularities in the obstacles. In order to facilitate the establishment of the model, this paper adopts the following provisions when establishing the obstacle model:

1. When the size of the area occupied by an obstacle on a unit grid is less than one grid, it is treated as occupying the entire area of the grid.
2. When there is a depression in the obstacle, fill the depression according to the grid.
3. Projecting the three directions of *XYZ* of the obstacle in the three-dimensional environment, so as to determine the position of the grid occupied by the obstacle. The predicted value is shown in Figure 9.
4. This plug-in is implemented in the environment of Maya7.0 using the built-in scripting language Mel. The main function of the plug-in is to simulate 2D plane or 3D plane group path planning. The plug-in interface details as follows:
5. Control the initial position of the particles to create two-dimensional plane particles or three-dimensional space particles.
6. The roles move to the center position due to mutual influence to generate the cluster behavior path.
7. The character can move according to the target point to generate a path following the leader.
8. The character will automatically bypass the obstacles ahead and generate an obstacle avoidance path.

Bind the human skeleton to the path generated by the Gaussian mutation algorithm, fine-tune the behavior of each individual, and add some random changes to the motion of each bound individual to create a group animation that is close to the desired effect—animators. The workload can be greatly reduced and attention can be turned to the design of the main characters and the design of the scene.

## 4. Conclusion

With the rapid development of computer graphics, 3D animation has been applied to all fields of people's lives, especially in the industries of film and television works, games, and entertainment. The wide application of animation technology makes it difficult for general 3D animation effects to impress increasingly discerning audiences. Group animation, as a new focus, has received more and more attention and has become a hot issue in computer graphics research. Traditional animation production mainly relies on manual drawing and key frame technology. The limitations of these technologies make the production of group animation consume a lot of manpower, financial resources, and time, and cannot guarantee the intelligence of characters and the authenticity of group behavior. Therefore, in order to avoid the above problems, this paper introduces an animation model generation method based on Gaussian mutation genetic algorithm optimization neural network, including obtaining animation scene data; according to the animation scene data, extracting animation model elements. The elements are input into the model-forming network, and the target animation model is generated: the target animation model is displayed. The method proposed in this paper improves the animation model generation method in the prior art to a certain extent. The animation model is constructed only through fixed rules, and the composition rules of the animation model cannot be changed according to the historical data of the animation model construction and the changes of other factors. Technical issues that reduce the flexibility and accuracy of animation model generation.

## Data Availability

The data used to support the findings of this study are available from the corresponding author upon request.
